# Solar-Driven Unmanned Hazardous and Noxious Substance Trapping Devices Equipped with Reverse Piloti Structures and Cooling Systems

**DOI:** 10.3390/polym14030631

**Published:** 2022-02-07

**Authors:** Ye Jin Kim, Hee Ju Kim, Yu Jin Seo, Ji Hee Choi, Hye Young Koo, Won San Choi

**Affiliations:** 1 Department of Chemical and Biological Engineering, Hanbat National University, 125 Dongseodaero, Yuseong-gu, Daejeon 305-719, Korea; agoqkfkrl@naver.com (Y.J.K.); kimhj0924@naver.com (H.J.K.); 6285999@naver.com (Y.J.S.); wlgml1350@naver.com (J.H.C.); 2 Functional Composite Materials Research Center, Jeonbuk Institute of Advanced Composite Materials, Korea Institute of Science and Technology (KIST), 92 Chudong-ro, Bongdong-eup, Wanju-gun, Seoul 136-791, Korea

**Keywords:** oil/water separation, absorbents, hazardous and noxious substance, evaporation

## Abstract

A solar-driven unmanned hazardous and noxious substance (HNS) trapping device that can absorb, evaporate, condense, and collect HNSs was prepared. The HNS trapping device was composed of three parts: a reverse piloti structure (RPS) for absorption and evaporation of HNSs, Al mirrors with optimized angles for focusing light, and a cooling line system for the condensation of HNSs. The RPS was fabricated by assembling a lower rectangle structure and an upper hollow column. The lower rectangular structure showed a toluene evaporation rate of 6.31 kg/m^2^ h, which was significantly increased by the installation of the upper hollow column (11.21 kg/m^2^ h) and led to the formation of the RPS. The installation of Al mirrors on the RPS could further enhance the evaporation rate by 9.1% (12.28 kg/m^2^ h). The RPS system equipped with an Al mirror could rapidly remove toluene, xylene, and toluene–xylene with high evaporation rates (12.28–8.37 kg/m^2^ h) and could effectively collect these substances with high efficiencies (81–65%) in an unmanned HNS trapping device. This prototype HNS trapping device works perfectly without human involvement, does not need electricity, and thus is suitable for fast cleanup and collection of HNSs in the ocean.

## 1. Introduction

Ocean pollution has continuously occurred worldwide via industrial wastewaters, daily life activities, and oil spill accidents [1,2]. Spill accidents frequently occur in the ocean during the transportation of oil. Oil, as well as hazardous and noxious substances (HNSs), can be spilled by spill accidents. An HNS can be defined as any substance that is likely to create hazards to marine ecosystems and human health [3]. HNSs transported by sea account for 11% of the chemicals traded worldwide, and since the carriage amount has increased by 3.5 times in the past 20 years, the risk of HNS spillage has further increased [4]. HNS spills are different from oil spills with regard to several aspects. HNS spills may be more disastrous than oil spills for both ecosystems and humans because HNSs, such as benzene (B), toluene (T), and xylene (X), are highly flammable and hazardous materials and, if evaporated into the air, provoke severe air pollution [5,6,7]. Thus, a different approach from oil/water separation or oil spills is needed for cleanup of HNS.

A variety of oil/water separation techniques, including conventional and advanced methods, have been proposed to address oil spill accidents. There are two types of typical approaches for oil/water separation: filtration and absorption. The filtration method is suitable for industrial oil/water separation settings because it allows a higher flux than the absorption method [8,9,10,11,12,13,14,15,16,17]. Since absorbents can be simply placed on oil spill sites and oil can be easily removed in situ by absorbents, the absorption method is more practical than the filtration method in cases of oil spill accidents [18,19,20,21,22,23,24,25,26,27,28]. For oil/water separation, a good absorbent should satisfy the following criteria [29,30]: (i) the absorbent should possess superhydrophobic and superoleophilic characteristics to selectively absorb oil from an oil/water mixture and (ii) the absorbent should possess a high specific surface area to absorb a large amount of oil. However, the currently available absorbents for oil/water separation may not have the necessary properties to be used for the absorption of HNS. In terms of materials, superhydrophobic or hydrophobic coatings can be dissolved or damaged by good solvents, such as BTX, upon short or long contact because most of the reported absorbents show a superhydrophobic or hydrophobic nature [29,30]. Highly cross-linked absorbents are free of dissolution or damage by BTX, but they are swelled and deformed [31]. The swelled or deformed absorbents are no longer available for oil or HNS cleanup. From a structural point of view, the reported absorbents have simple structures because they only handle absorption missions [29,30], while the HNS absorbents deal with absorption and evaporation missions. Based on the absorption–evaporation process of HNSs, the absorption function alone is insufficient for HNS harvesting devices. Compared to the reported absorbents for oil/water separation, the absorbent for HNSs should possess different characteristics in terms of materials and structures. Thus, studying the material and structure of HNS absorbents is necessary for the effective absorption and evaporation of HNSs.

Most of the reported oil/water separation methods are manned. However, HNS spill control work prohibits human involvement due to the highly flammable and hazardous nature of HNSs [5,6]. The only way to clean up HNSs is by using a fence to prevent leakage and then natural evaporation, which leads to the secondary contamination of air [5,6]. Moreover, this natural evaporation method is inefficient and uneconomical because expensive HNSs, such as BTX, cannot be collected. Thus, it is necessary to develop techniques for the simultaneous cleanup and collection of HNSs in the absence of manpower. To the best of our knowledge, the unmanned cleanup and collection of HNSs in the ocean has not yet been reported.

Herein, we report a solar-driven unmanned HNS trapping device loaded with a reverse piloti structure (RPS) and cooling system that can absorb, evaporate, condense, and collect HNSs. This prototype unmanned device can be used for cleanup and collection of valuable HNSs thanks to the hydrophilic RPS that possesses excellent dissolution and swelling resistance against HNSs.

## 2. Experimental Section

### 2.1. Materials

A polyurethane sponge (PUS) was purchased from Allfoam (Seoul, Korea). A candle was purchased from Daekwang (Seoul, Korea). Ethylene glycol (C_2_H_6_O_2_, 99.8%), sodium acetate (CH_3_COONa, ≥99.0%), iron(III) chloride hexahydrate (FeCl_3_·6H_2_O, ≥98.0%), polyvinyl alcohol (Mw = 130,000 and 13,000 Da, 99.0% hydrolyzed), ethanol (EtOH, 96.5%), toluene (C_6_H_5_CH_3_, 99.5%), xylene (C_6_H_5_(CH_3_)_2_, 99.0%), dopamine hydrochloride (100%), and acetone ((CH_3_)_2_CO, 99.0%) were purchased from Sigma-Aldrich. Konjac glucomannan (KGM) was purchased from Zhejiang. Polyester thread was purchased from Matata (Seoul, Korea). All chemicals were used without further purification. Deionized (DI) water with a resistance of 18 MΩ cm was obtained from a Millipore Simplicity 185 system.

### 2.2. Preparation of the Magnetic Nanoparticles (MNPs)

FeCl_3_·6H_2_O (0.9 g) and CH_3_COONa (2.4 g) were dissolved in ethylene glycol (30 mL) under vigorous stirring for 5 min at room temperature. The resulting solution was transferred to a Teflon-lined autoclave and heated in an oven at 200 °C for 8 h. The final product was washed three times with ethanol and DI water and dispersed in DI water for further use.

### 2.3. Preparation of CS

Carbon soot (CS) nanoparticles were prepared by a combustion flame process in open air using a candle. The CS powder was collected by placing a silicon wafer on top of the flame.

### 2.4. Preparation of PUS/MNP/PVA

The MNPs were dispersed in DI water (6.5 mg/500 μL) by ultrasonication. The lower rectangle structure (PUS, 2 × 2 × 1 cm^3^) or hollow column (PUS, inner diameter: 0.2 cm, outer diameter: 0.5 cm, and length: 2 cm) was immersed in the resulting solution and sonicated for 1 h. The resulting rectangle or hollow column structure (PUS/MNP) was dried in an oven at 50 °C without a rinsing step for 3 h. Each structure (PUS/MNP) was washed three times with DI water and dried in an oven at 50 °C for 4 h. Polyvinyl alcohol (PVA) powder (0.1, 1, or 2 g) was dissolved in 100 mL of DI water while stirring at 180 °C for 2 h. Each structure (PUS/MNP) was immersed in the resulting PVA solution for 1 h and heated in an oven at 100 °C for 3 h.

### 2.5. Preparation of PUS/CS/PVA

Five milligrams of the CS nanoparticles were sonicated in 3 mL of acetone for 1 h to disperse the CS particles. The PUS (2 × 2 × 1 cm^3^) was immersed in the resulting CS solution for 5 s and then removed. The resulting PUS was dried in an oven at 50 °C for 30 min. The procedure for coating PVA was the same as the abovementioned method.

### 2.6. Preparation of Polyester Thread/PVA-KGM

PVA powder (Mw = 130,000 Da, 5 g) was dissolved in 95 mL of deionized water under stirring at 180 °C for 3 h. Then, KGM powder (0.28 g) was dissolved in the resulting solution at 100 °C for 2 h. The polyester thread (D: 3 mm, L: 35 cm) was immersed in the resulting solution for 1 h and heated in an oven at 100 °C for 1 h without washing.

### 2.7. HNS Evaporation

All HNS evaporation tests were performed at room temperature (~25 °C) for 30 min at a humidity of 45–50%. Each sample was tested three times, and the average value was used. A xenon lamp was used for visible light irradiation. The experiment was conducted with a distance of 60 cm between the sample and the lamp. The sample was fixed to a beaker that contained 30 mL DI water and 10 mL HNS. The weight of the resulting beaker before and after the evaporation test was measured on an electronic balance. The hollow space between the sample and beaker was covered with an aluminum plate to prevent evaporation from the HNS surface.

### 2.8. Characterization

Scanning electron microscopy (SEM) and energy dispersive X-ray (EDX) analyses were carried out using a JEOL JEM 2100F system and Hitachi S-4800 system, respectively. Thermogravimetric analysis (TGA) was performed using a thermogravimetric analyzer (Sinco TGA N-1500) over a temperature range of 25–800 °C at a heating rate of 5 °C min^−1^ in air (flow rate, 60 cm^2^ min^−1^). X-ray diffraction (XRD) patterns were obtained on a Rigaku X-ray diffractometer equipped with a Cu Kα source. Water contact angle (WCA) measurements were carried out using a contact angle meter (SEO Phoenix 300 Touch) at ambient temperature, and the volume of the probing liquid was 20 μL. Fourier transform infrared (FT-IR) spectra were obtained using a Sinco Nicolet IS5 instrument. Visible light was irradiated on the sample using a xenon lamp (Spectro, XLS-300P) with a light power density of 0.3 kW m^−2^. The weight of the sample before and after the evaporation test was measured on an electronic balance (Sartorius BSA224S). An electric rpm-controlled DC motor (3.7 V, Ntrex, Incheon, Korea) equipped with a fan was used to create a constant air velocity of the air flow, which was injected into the sample. After the injection of the air flow, the pressure drop was measured at the inlet and outlet of the sample using a differential pressure gauge (TESTO 510i, TESTO, Titisee-Neustadt, Germany), and the air velocity was measured at the inlet and outlet of the sample using a flowmeter (TESTO 450i, TESTO, Titisee-Neustadt, Germany).

## 3. Results and Discussion

**Figure 1:**
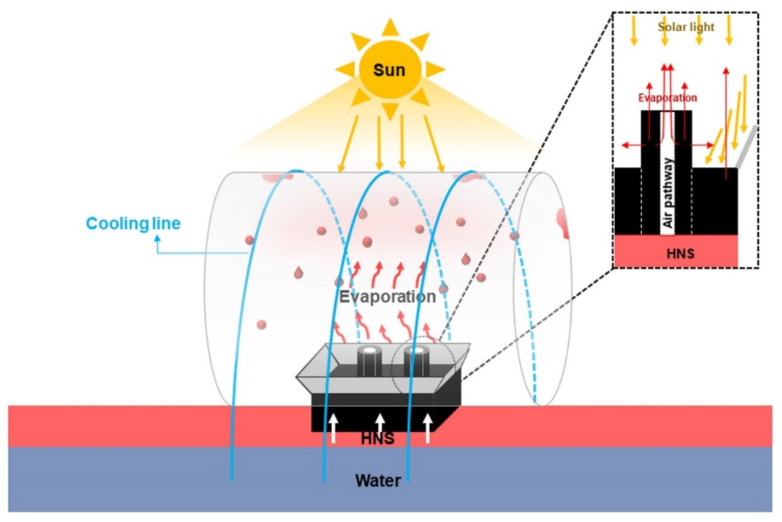
Schematic illustration of an HNS trapping device consisting of the RPS for absorption and evaporation of HNSs, an Al mirror for focusing light, and a cooling system for condensation of HNSs.

The HNS harvesting device was composed of three parts: an RPS for absorption and evaporation of HNSs, an Al mirror for focusing light, and a cooling line-coated glass dome for HNS condensation (Figure 1). An RPS was fabricated by assembly of the lower rectangle (2 × 2 × 1 cm^3^) and upper hollow column (inner diameter: 0.2 cm, outer diameter: 0.5 cm, and length: 2 cm) structures that were prepared by the stepwise coating of nanoparticles (NPs) and PVA on a PUS. Two types of RPSs (RPS-MNP: PUS/MNP/PVA and RPS-CS: PUS/CS/PVA) were prepared by coating MNPs and PVA and CS and PVA on a PUS. PUS with porous structures was employed as a main evaporation route for HNSs. MNPs (or CS) played as a role for enhancing the specific surface area. PVA was used as a binder and hydrophilic component. An Al mirror with an optimized angle was installed at the top of the RPS to maximize the absorption of the light. The cooling lines (polyester thread/PVA-KGM) were coated on the rooftop of the glass dome to effectively condense the HNS vapor. The hydrophilic cooling line contained a certain amount of water and kept the rooftop cooler than the surroundings because both ends of the cooling line were immersed in water to absorb and transfer the water.

**Figure 2:**
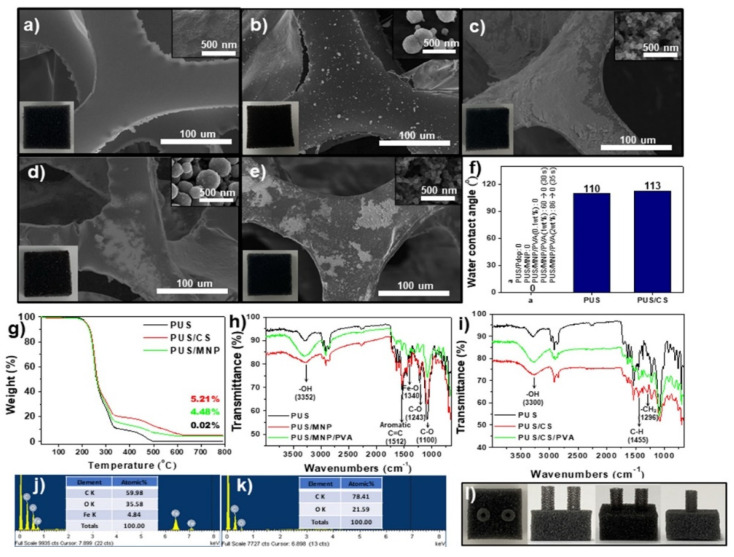
SEM data for the (**a**) PUS, (**b**) PUS/MNP, (**c**) PUS/CS, (**d**) PUS/MNP/PVA, and (**e**) PUS/CS/PVA. (Inset) Corresponding images of (**a**–**e**). (**f**) WCAs of various samples. (**g**) TGA data for PUS, PUS/CS, and PUS/MNP. FT-IR data for the (**h**) PUS/MNP/PVA and (**i**) PUS/CS/PVA. EDX data for the (**j**) PUS/MNP/PVA and (**k**) PUS/CS/PVA. (**l**) Images of the RPSs.

Figure 2a–e shows SEM images of the formation processes of the two types of RPSs. The PUS showed an interconnected 3D network skeleton with a smooth surface morphology (Figure 2a). After MNP and CS coating of the PUS, the surfaces of the PUS were covered with MNPs and CS, which had average sizes of 230 nm and 55 nm, respectively (Figure 2b,c and Figure S1). RPS-MNPs (PUS/MNP/PVA) and RPS-CS (PUS/CS/PVA) were prepared by PVA coating on PUS/MNPs and PUS/CS, respectively (Figure 2d,e). After PVA coating, each MNP or CS particle tended to be more agglomerated in the PUS/MNP (or CS)/PVA sample than in the PUS/MNP sample, which indicated that each particle mixed with PVA, moved, and reprecipitated during PVA coating. Some MNP and CS fragments were detached from the PUS/MNP and PUS/CS upon handling, respectively, but after PVA coating, no fragments detaching from the RPS-MNP and RPS-CS were observed. No remarkable changes in the color of the RPS-MNPs and RPS-CS were observed compared to that of the PUS (inset of Figure 2a–e). The WCAs on the PUS, PUS/MNP, PUS/polydopamine (Pdop), and PUS/CS were 110°, 0°, 0°, and 113°, indicating that the PUS, PUS/MNP, PUS/Pdop, and PUS/CS possessed hydrophobic, superhydrophilic, superhydrophilic, and hydrophobic characteristics, respectively (Figure 2f). After PVA coating (0.1, 1, and 2 wt%), the WCAs of PUS/MNP/PVA and PUS/CS/PVA were 0° (all cases), indicating a superhydrophilic nature (Figure 2f). Several measurements were performed to confirm the formation of two types of RPSs. The amounts of MNPs and CS loaded onto RPS-MNPs and RPS-CS, respectively, were measured by TGA, indicating that the MNPs and CS accounted for 4.48% and 5.21% of RPS-MNPs and RPS-CS, respectively (Figure 2g). After coating of MNPs and CS onto PUS, the thermal stability of PUS/MNPs and PUS-CS slightly enhanced in the range of 300–600 °C due to the presence of MNPs and CS, respectively (PUS/CS > PUS/MNPs > PUS). The absorption peaks at 1512 cm^−1^ (aromatic C=C), 1243 cm^−1^ (C-O), and 1100 cm^−1^ (C-O), the characteristic groups in PU, were decreased due to the coating of MNPs for PUS/MNPs (red line, Figure 2h) [32]. New absorption peaks at 1340 cm^−1^ (Fe-O) were also observed for PUS/MNPs (red line) [33]. The intensities of peaks at 1455 cm^−1^ and 1296 cm^−1^ related to aliphatic -CH and -CH_2_ significantly increased for PUS/CS (red line) (Figure 2i) [32]. After PVA coating on the PUS/MNPs and PUS/CS, the absorption peaks at 3352 cm^−1^ and 3300 cm^−1^ (-OH) originating from PVA increased and became strong (green line, Figure 2h,i) [32]. As shown in the EDX data, Fe was observed in the RPS-MNPs (Figure 2j). The content of C in RPS-CS was much higher than that of C in RPS-MNPs (Figure 2k). XRD data showed the typical XRD patterns of MNPs (magnetite, Fe_3_O_4_) (JCPDS 19-629), which indicated that the MNPs had been incorporated into RPS-MNPs (Figure S2). The abovementioned data suggested that the PUS had been successfully coated with layers of MNP/PVA or CS/PVA to form RPS-MNPs or RPS-CS, respectively.

**Figure 3:**
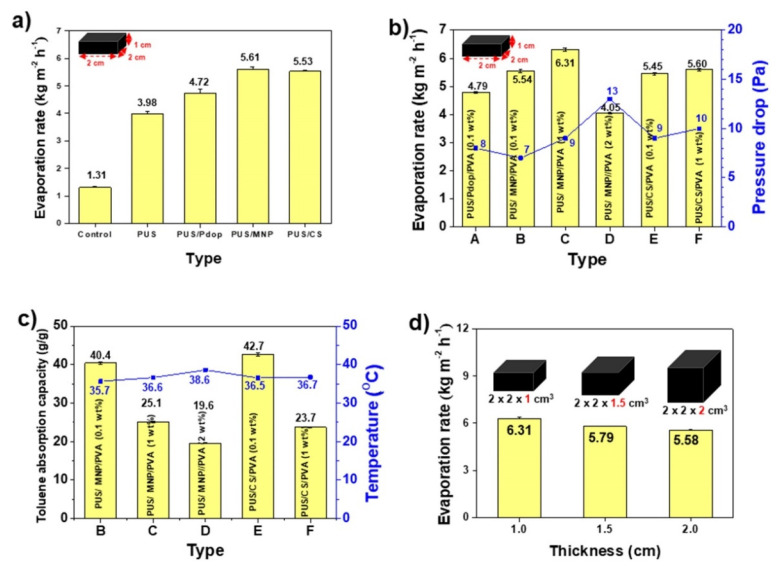
(**a**) Toluene evaporation rates for rectangular structures made of PUS, PUS/Pdop, PUS/MNP, and PUS/CS before coating PVA. (**b**) Toluene evaporation rates and pressure drops for the abovementioned samples after coating PVA with 0.1–2 wt%. (**c**) Toluene absorption capacity and temperature of rectangular structures made of PUS/MNP/PVA (0.1–2 wt%) and PUS/CS/PVA (0.1–1 wt%). (**d**) Toluene evaporation rates of rectangular structures made of PUS/MNP/PVA (1 wt%) with different thicknesses of 1–2 cm.

Since the RPS consisted of upper hollow column and lower rectangle structures, the toluene evaporation rate of individual structures was investigated. First, the lower rectangle structure (2 × 2 × 1 cm^3^) made of PUS/MNP/PVA or PUS/CS/PVA was tested under visible light illumination at a light power density of 0.3 kW m^−2^. For comparison, 4 rectangular structures made of bare PUS, PUS/Pdop, PUS/MNP, or PUS/CS were also tested. The evaporation rate of PUS/CS was higher than that of PUS, but they possessed analogous WCAs (110° and 113°) on the PUS and PUS/CS, respectively, which suggested that the rough surface of the PUS/CS could effectively evaporate toluene (Figure 3a and Figure 2c). Although the PUS/Pdop and PUS/MNP cases had an identical surface nature, such as superhydrophilicity, PUS/MNP with a rough surface showed a higher evaporation rate than PUS/Pdop (Figure 3a). Interestingly, superhydrophilic PUS/MNP showed better performance than hydrophobic PUS/CS for the evaporation of toluene (Figure 3a). Upon handling of PUS/MNP and PUS/CS, the detachment of some fragments of MNP and CS was observed. To prevent detachment of fragments, various polymers were tested for coating materials. Hydrophobic polymers such as polystyrene (PS), polymethylmethacrylate (PMMA), polydecylmethacrylate (PDMA), polybutylmethacrylate (PBMA), and cross-linked polydimethylsiloxane (PDMS) were coated on PUS/MNPs and PUS/CS. However, these polymers were completely dissolved in toluene and xylene (data not shown). Furthermore, after dissolution of the coating materials, the PUS (cross-linked product) was swelled and deformed by toluene and xylene (Figure S3). The sizes of the PUS were increased by 180% and 136% in toluene and xylene, respectively. These results indicated that hydrophobic and even cross-linked polymers were not suitable for coating materials and BTX evaporation. Thus, to prevent dissolution and swelling of coating materials and PUS, hydrophilic polymers, such as KGM and PVA were coated on PUS/MNP and PUS/CS. Deacetylated KGM was partially dissolved and detached in toluene and xylene after several uses (data now shown). However, PVA (molecular weight (MW): 130,000 Da) was not dissolved even after reuse over 25 cycles and prevented the PUS from swelling in toluene and xylene (Figure S3). To further confirm the dissolution and swelling resistances of PVA, PVA film (1 wt%) was prepared and immersed in toluene at 45 °C for 2 h. No changes in weight or surface morphology of the PVA film were observed before and after immersion of the PVA film in toluene (Figure S4). Hydrophobic and cross-linked polymers that can be dissolved and swelled in BTX are not suitable for BTX evaporation because BTX is a good solvent for most hydrophobic polymers. However, PVA showed excellent dissolution and swelling resistances against toluene and xylene because BTX was a poor solvent for hydrophilic PVA possessing repeating hydroxyl groups. Since the coating of PVA could prevent the detachment of fragments and protect the PUS from swelling, PVA was selected for coating materials and BTX evaporation.

To investigate the effect of PVA content on the evaporation rate, PVA with different contents (0.1, 1, and 2 wt%) was coated on PUS/MNPs and PUS/CS. After PVA coating, no fragments or swelling were observed upon handling the rectangular structure (PUS/MNP(or CS)/PVA) in all cases. As the PVA content increased, the evaporation rate increased except for the case of 2 wt% (Figure 3b). Performance reduction was observed for the PUS/MNP/PVA (2 wt%) sample due to the reduction in the internal volume of the rectangular structure by PVA coating with a high content. Among the samples (0.1–2 wt%), the PUS/MNP/PVA (1 wt%) sample showed the best toluene evaporation rate (6.31 kg/m^2^ h), which was increased value (113%) compared to the PUS/MNP (Figure 3a,b). To reveal the reason for the best performance of PUS/MNP/PVA (1 wt%), the toluene absorption capacities of the samples were measured. The absorption capacity means the maximum amount that each sample can absorb. The absorption capacities of the PUS/MNP/PVA (0.1, 1, and 2 wt%) samples were 40.4, 25.1, and 19.6 g/g, respectively (Figure 3c). As the PVA content increased, the toluene absorption capacity decreased because the internal volume of the rectangular structure decreased. These results could explain the performance reductions for the cases of 2 wt%. The absorption capacity (25.1 g/g) of the PUS/MNP/PVA (1 wt%) sample showing the best evaporation rate was lower than that (40.4 g/g) of the PUS/MNP/PVA (0.1 wt%) sample, which suggested that a small amount of toluene could be efficiently evaporated compared to a large amount of toluene (Figure 3b,c). However, the PUS/MNP/PVA (2 wt%) sample possessing the lowest absorption capacity (19.6 g/g) did not show the best evaporation rate.

To reveal the reason, the pressure drops of the samples were measured. The pressure drop can be defined as the difference in pressure of a fluid between two points. Generally, the pressure drop increases when a fluid passes through complicated structures, preventing fluid flow. The PUS/MNP/PVA (2 wt% and 0.1 wt%) samples showed the highest (13 Pa) and lowest (7 Pa) pressure drops, respectively, which suggested that toluene vapor hardly and easily penetrated through the sample (2 wt% and 0.1 wt%), respectively (Figure 3b). In other words, although the PUS/MNP/PVA (2 wt%) sample contained a small amount of toluene for efficient or easy evaporation, it had an unfavorable intra-structure for toluene evaporation due to the high pressure drop. Although the PUS/MNP/PVA (0.1 wt%) sample had a favorable intra-structure for the evaporation of toluene due to the low pressure drop, it contained a large amount of toluene. Thus, effective evaporation of toluene was not observed in either PUS/MNP/PVA (2 or 0.1 wt%) sample for these reasons. These results indicated that two parameters, namely, absorption capacity and pressure drop, should be simultaneously considered for efficient evaporation of toluene. To find another parameter affecting the evaporation rate, the surface temperatures of the samples were measured under visible light illumination. Temperatures measured at five points of each sample were used for average surface temperatures. The average surface temperatures varied. The PUS/MNP/PVA (0.1 and 2 wt%) samples possessing the highest and lowest toluene absorption capacities showed the lowest (35.7 °C) and highest (38.6 °C) surface temperatures due to the difference in the toluene amount, respectively (Figure 3c). As the toluene absorption amount increased, the surface temperature decreased. These results suggested the two following facts. First, a large amount of toluene as well as a low surface temperature could disturb the efficient evaporation of toluene at the surface of the structure (PUS/MNP/PVA (0.1 wt%)). Second, a high pressure drop of the structure could also disturb the efficient evaporation of toluene even though the structure contained a small amount of toluene and possessed a high surface temperature (PUS/MNP/PVA (2 wt%)). The PUS/MNP/PVA (1 wt%) sample showing the best evaporation rate possessed a medium surface temperature (36.6 °C) (Figure 3b,c). Although the highest and lowest values of each parameter were not helpful for the enhancement of evaporation rate, the pressure drop, absorption capacity, and temperature simultaneously and complexly affected the evaporation rate. Thus, the abovementioned results suggested that the sample should possess an appropriate absorption capacity (25.1 g/g), pressure drop (9 Pa), and surface temperature (36.6 °C) that are not too high or low for efficient evaporation. The PUS/CS/PVA (1 wt%) sample with the 2nd highest evaporation rate showed analogous absorption capacity (23.7 g/g), pressure drop (10 Pa), and surface temperature (36.7 °C) to PUS/MNP/PVA (1 wt%), which further supported the abovementioned results (Figure 3b,c). Thus, according to the abovementioned results, the PUS/MNP/PVA (1 wt%) sample was determined to be an optimized condition for the lower rectangle structures.

The effect of the MW of PVA on the dissolution resistance and evaporation rate was investigated. To evaluate the high MW (HMW)-PVA (130,000 Da) used in this study, a low MW (LMW)-PVA (13,000 Da) film was prepared and compared to the dissolution resistance in toluene. The weight and surface morphology of the LMW-PVA film slightly decreased and changed after immersion of the LMW-PVA film in toluene, respectively, which was not observed in the HMW-PVA film (Figure S5). We speculated that compared with LMW-PVA, HMW-PVA showed a higher dissolution resistance against organic solvents such as toluene because it possessed many more hydroxyl groups to prevent toluene invasion than LMW-PVA. The low dissolution resistance of LMW-PVA resulted in performance degradation. The PUS/MNP/LMW-PVA sample showed a lower evaporation rate (5.83 kg/m^2^ h) than the PUS/MNP/HMW-PVA (6.31 kg/m^2^ h) (Figure S6). Thus, HMW-PVA was employed in our study. The evaporation rates of the rectangular structure with different thicknesses were also measured to investigate the thickness effect on the evaporation rate. As the thickness of the structure increased (1, 1.5, and 2 cm), the evaporation rate of the structure decreased (Figure 3d). It was not easy to transfer enough toluene to the top surface from the bottom of the rectangular structure as the thickness of the rectangular structure increased due to the low surface tension of toluene (28.52 Nm^−1^ at 20 °C). Thus, PUS/MNP/HMW-PVA (1 wt%) (2 × 2 × 1 cm^3^) with a thickness of 1 cm was determined to be an optimized condition for the lower rectangular structures. A rectangular structure with thicknesses below 1 cm (0.5 cm) was excluded because it had a structure that was too thin to support the upper hollow columns.

**Figure 4:**
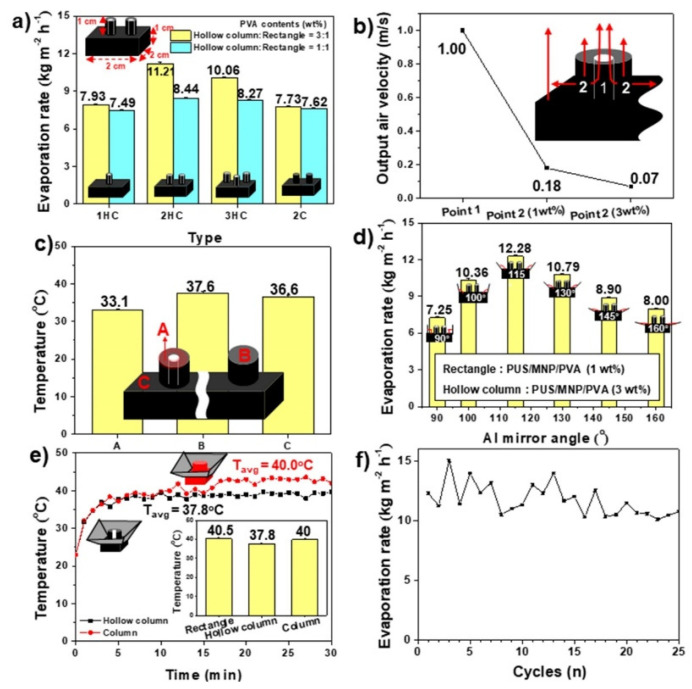
(**a**) Toluene evaporation rates for the RPSs with different numbers of hollow columns (n: 1–3). The RPS samples consisted of rectangular structures [PUS/MNP/PVA (1 wt%)] and hollow columns [PUS/MNP/PVA (1 wt% and 3 wt%)]. (**b**) Output air velocity and (**c**) surface temperature data at certain points of the RPS samples composed of rectangular structures [PUS/MNP/PVA (1 wt%)] and hollow columns (PUS/MNP/PVA (1 and 3 wt%)). Each inset shows an illustration of the RPS showing a certain point. (**d**) Toluene evaporation rates for RPSs equipped with Al mirrors at different angles. (**e**) Surface temperature data of hollow column and column-equipped RPS samples with Al mirrors. Inset shows the average temperature. (**f**) Long-term performance of the optimized RPS sample with Al mirrors.

To further enhance the evaporation rate of the rectangular structure, the upper hollow column structure made of PUS/MNP/PVA (1 wt%, MW = 350,000 Da) was installed on the rectangular structure (PUS/MNP/PVA (1 wt%, MW = 350,000 Da), which led to the formation of the RPS. Three types of RPSs installed with 1–3 hollow columns were tested. The evaporation rates of the RPS with 1 and 2 hollow columns increased to 7.49 and 8.44 kg/m^2^ h, respectively, which were much higher than the 6.31 kg/m^2^ h of the rectangular structure without a hollow column structure (ice blue bars, Figure 4a). The RPS with 2 hollow columns showed the best performance, while the RPS with 3 hollow columns exhibited a decreased evaporation rate (8.27 kg/m^2^ h). To reveal the reason why the RPS with 2 hollow columns showed outstanding performance, the RPS with 2 columns was prepared and compared. The columns installed on the rectangular structure were nonhollow structures. This RPS with 2 columns showed a lower evaporation rate (7.62 kg/m^2^ h) than the RPS with 2 hollow columns (8.44 kg/m^2^ h) (ice blue bars, Figure 4a). The toluene at point 2 can be moved to the top surface and evaporated through the internal porous structure of the hollow column (inset of Figure 4b). According to Bernoulli’s principle, the toluene at point 2 could also move to the hollow space (point 1) of the hollow column to be evaporated by pressure differences at points 1 and 2. To confirm this, the output air velocities at the 2 points were measured under a constant input air velocity of 1 m/s. The output air velocity at point 1 was 1 m/s, which was much faster than that (0.18 m/s) at point 2 (Figure 4b). Bernoulli’s principle states that as the velocity of a fluid increases, the pressure of a fluid decreases simultaneously [34]. Thus, low and high pressures could be formed at points 1 and 2, respectively, which could promote the movement of toluene vapor at point 2 to the hollow space (point 1) of the hollow column. Thus, this toluene vapor can be easily evaporated through the hollow channel (point 1) of the hollow column. We speculated that the hollow column structure created a high difference in pressure within the hollow column, which induced the active circulation of toluene vapor for efficient evaporation.

To further confirm this hypothesis, we designed denser hollow columns by increasing the PVA content. Denser hollow columns made of PUS/MNP/PVA (3 wt%) than PUS/MNP/PVA (1 wt%) were prepared for a large difference in pressure. The output air velocity of the hollow column (PUS/MNP/PVA (3 wt%)) was 0.07 m/s, which was lower than that (0.18 m/s) of the hollow column (PUS/MNP/PVA (1 wt%)) (Figure 4b). As a result, the 3 wt% case had a larger difference in velocity than the 1 wt% case (△v = 0.93 m/s and 0.82 m/s for 3 wt% and 1 wt% cases, respectively). Thus, a larger difference in pressure was formed in the case (3 wt%) than in the case (1 wt%). In other words, the hollow column (PUS/MNP/PVA (3 wt%)) possessed a larger difference in pressure between points 1 and 2 than the hollow columns (PUS/MNP/PVA (1 wt%)) because the larger the difference in velocity is, the larger the difference in pressure. Actually, the RPS with 2 hollow columns (PUS/MNP/PVA (3 wt%)) showed a much higher evaporation rate (11.21 kg/m^2^ h) than the RPS with 2 hollow columns (PUS/MNP/PVA (1 wt%)) (8.44 kg/m^2^ h) (yellow bars, Figure 4a). The other cases showed the same phenomena. These results further confirmed that the large difference in the pressure within the hollow column could create active circulation of toluene vapor for effective evaporation. The PVA coating over 4 wt% caused the deformation of the hollow column due to the high content of PVA (data not shown). The temperature difference between the top surfaces of the hollow column and the rectangular structure could also contribute to the active circulation of toluene vapor. The top surface of the hollow column showed a much lower temperature (33.1 °C) than that of the column (37.6 °C) (Figure 4c). Thus, the temperature difference (△T = 3.5 °C) at the top surfaces of the hollow column (33.1 °C) and rectangular structure (36.6 °C) was larger than that (△T = 1 °C) of the column (37.6 °C) and rectangular structure (36.6 °C), which further accelerated the active circulation of toluene vapor within the hollow column (Figure 4c). Thus, the RPS with 2 hollow columns, a rectangular structure and a hollow column made of PUS/MNP/PVA (1 wt% and 3 wt%, respectively), were determined to be an optimized RPS for the absorption and evaporation of HNSs.

To enhance the absorption of light by the RPS, 4 Al mirrors with different angles of 90–160° were installed around the RPS with 2 hollow columns. The fundamental role of the Al mirrors is to collect and focus light on the RPS. To find the optimized angle of the Al mirror, 6 RPS samples with different Al mirror angles were tested (Figure S7). Among the 6 angles, only the RPS sample with an Al mirror angle of 115° showed a higher evaporation rate (12.28 kg/m^2^ h) than the RPS without an Al mirror (11.21 kg/m^2^ h), indicating that only a certain angle of the Al mirror could efficiently collect and focus light on the RPS (Figure 4d). Thus, 115° was determined to be the optimized angle of the Al mirror. The temperature of the rectangular structure was increased to 40.5 °C by Al mirrors (Figure 4e). The temperatures of the hollow column and column also increased to 37.8 °C and 40 °C, respectively (Figure 4e). However, the temperature difference (2.7 °C) at the top surfaces of the hollow column (37.8 °C) and rectangular structure (40.5 °C) was larger than that (0.5 °C) of the column (40 °C) and rectangular structure (40.5 °C), which was in accord with the RPS results in the absence of Al mirrors (Figure 4e). The active circulation of toluene vapor could still be maintained by a large difference in the temperature between the rectangular structure and hollow column of the RPS with Al mirrors. Although high temperature is helpful for the evaporation of toluene, exposure of flammable BTX to high temperature may present a fire risk. Thus, a system or structure to restrict an excessive temperature rise is necessary for BTX evaporators. We expect that the hollow column of the RPS could play an important role in vapor circulation as well as cooling tower because the hollow column could maintain a low temperature (△T = −2.7~−3.5 °C) around itself due to the heat of evaporation formed by evaporation of toluene (Figure 4c,e). To find an optimized hollow column ratio, hollow columns possessing higher (50%) and lower (30%) ratios than the hollow column ratio (40%) used in this study were prepared, installed on a rectangular structure, and tested. Among the samples, the hollow column case possessing a hollow ratio of 40% showed the highest evaporation rate, indicating that a certain hollow ratio of the hollow column was necessary for optimized evaporation (Figure S8). The long-term performance of the RPS with Al mirrors was evaluated for the practical application of the RPS. The evaporation rate of the RPS with Al mirrors was variable. However, the evaporation rates remained as high as 15.04–10.46 kg/m^2^ h for up to 25 cycles (Figure 4f).

**Figure 5:**
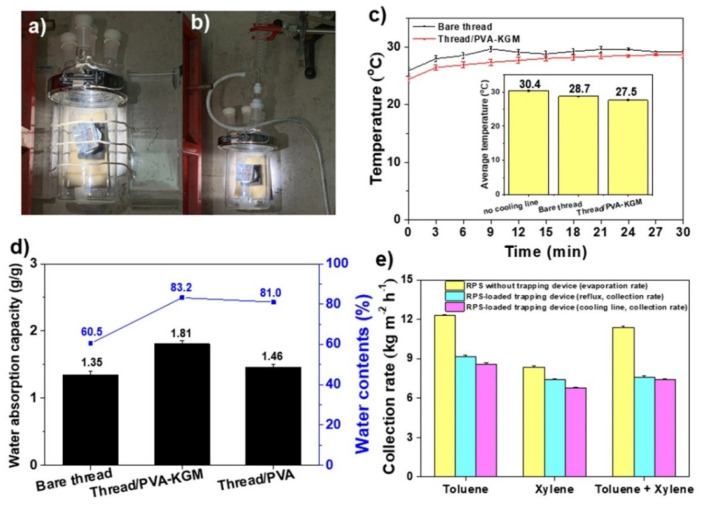
Images of the HNS trapping device (RPS equipped with an Al mirror) installed with (**a**) cooling lines and (**b**) a reflux condenser. (**c**) Temperatures of the rooftop of a glass chamber installed with cooling lines (polyester thread and polyester thread/PVA-KGM). (**d**) Water absorption capacity and water content of thread, thread/PVA, and thread/PVA-KGM. (**e**) Collection rates of the RPS-loaded HNS trapping device with different cooling systems for toluene, xylene, and toluene–xylene.

To investigate the possibility for a real application (collection of HNSs), the optimized RPS system (RPS equipped with an Al mirror) was placed in a glass chamber (1 L, HNS trapping device) equipped with cooling lines (cooling line system) or a reflux condenser (reflux system) (Figure 5a,b). Two cooling systems were employed to enhance the HNS trapping ratio. For a cooling line system, both ends of the cooling lines were immersed in water to absorb and transfer the water to the rooftop of the chamber. The cooling line (polyester thread/PVA-KGM) was prepared by coating a mixture of PVA and KGM on the polyester thread. The combination of PVA and KGM was used due to the strong mechanical properties of PVA and the excellent hydrophilicity of KGM. In the absence of cooling lines, the temperature of the rooftop of the glass chamber was 30.4 °C, while it was 28.7 °C in the presence of cooling lines (bare polyester thread), which was further decreased to 27.5 °C in the presence of cooling lines (polyester thread/PVA-KGM) (Figure 5c). To reveal the reason why the polyester thread/PVA-KGM sample could keep the rooftop of the glass chamber cooler than the other cooling lines, the water absorption capacity and water content of the thread, thread/PVA, and thread/PVA-KGM samples were measured. Among the samples, the thread/PVA-KGM sample exhibited the highest water absorption capacity (1.81 g/g), which was 134% and 124% of the water absorption capacity of the bare thread and thread/PVA samples, respectively (Figure 5d). The thread/PVA-KGM sample also maintained a water content over 83% even after illumination with visible light for 6 h, which was higher than the 60.5% and 79% water contents of the thread and thread/PVA samples, respectively (Figure 5d). The hydrophilic polyester thread/PVA sample can absorb more water than the hydrophobic polyester thread because of the PVA. However, the polyester thread/PVA-KGM sample can absorb and contain much more water than the polyester thread/PVA and polyester thread samples because KGM had 9–11 times more hydroxyl groups per repeating unit than PVA. PVA-KGM could capture water molecules through hydrogen bonds formed by numerous hydroxyl groups and prevent water molecules from evaporating. Thus, we speculated that the water-absorbing and water-containing abilities of PVA-KGM made a difference. The abovementioned results suggested that the PVA-KGM-coated cooling line could absorb, transfer, and maintain a sufficient amount of water to cool the rooftop of the glass chamber over the whole period of illumination. 

Before discussing the ability of the HNS trapping device equipped with different cooling systems, the evaporation rates of the RPS system without the HNS trapping device were measured for xylene and toluene/xylene mixtures. The xylene evaporation rate was lower than the toluene evaporation rate due to the higher molar mass and boiling point than toluene (X: 106.19 g/mol and 139 °C > T: 92.14 g/mol and 110 °C) (yellow bars, Figure 5e) [35]. The toluene/xylene mixture (1:1, *v*/*v*) evaporation rate was slightly lower than the toluene evaporation rate (yellow bars, Figure 5e). Although benzene was not tested due to its high toxicity, a higher evaporation rate of benzene is expected to be obtained because of its much lower molar mass and boiling point (B: 78.11 g/mol and 80 °C) than toluene and xylene [35]. The collection rates of the three types of HNSs were measured by an RPS-loaded HNS trapping device with different cooling systems. The toluene, xylene, and toluene/xylene collection rates of the HNS trapping device equipped with the cooling line were 8.6, 6.75, and 7.39 kg/m^2^ h, which were 70%, 81%, and 65% of the evaporation rate of the RPS system without the HNS trapping device, respectively (pink bars, Figure 5e). The reflux condenser system showed slightly higher collection rates (9.18, 7.39, and 7.61 kg/m^2^ h) than the cooling line system, which was 75%, 88%, and 67% of the evaporation rate of the RPS system without the HNS trapping device (ice blue bars, Figure 5e). No phase separation phenomena were observed in collected toluene, xylene, and toluene/xylene, indicating that only toluene or xylene was evaporated and collected. After evaporation test, water amount in toluene (or xylene)/water mixture was measured to confirm the possibility of evaporation of water. No change in amount of water was observed before and after evaporation test of toluene or xylene. Each system has advantages for real applications. Although a higher collection ratio can be achieved by using the reflux system if necessary, the HNS trapping device equipped with a cooling line system is closer to the real application because a significant amount of HNS can be collected by a nonpowered system. (1–4)

## 4. Conclusions

A solar-driven unmanned HNS trapping device loaded with RPS and a cooling system that can absorb, evaporate, condense, and collect HNS was prepared. The RPS was fabricated by assembly of the lower rectangle structure and upper hollow column. The lower rectangle structure showed a toluene evaporation rate of 6.31 kg/m^2^ h, which was significantly increased by installation of the upper hollow column (11.21 kg/m^2^ h) and led to the formation of the RPS. The installation of Al mirrors on the RPS could further enhance the evaporation rate by 9.1% (12.28 kg/m^2^ h). The hollow column of the RPS created large differences in pressure and temperature, which induced the active circulation of HNS vapor for efficient evaporation. The hollow column could also play an important role in the cooling tower to restrict an excessive temperature rise. The long-term performance of the RPS with Al mirrors remained as high as 15.04–10.46 kg/m^2^ h for up to 25 cycles. Thanks to the chemically stable and structurally optimized RPS for absorption and evaporation of HNS, Al mirrors with optimized angle for focusing of light, and cooling line for condensation of HNS, the RPS system equipped with Al mirror could rapidly remove toluene, xylene, and toluene-xylene with high evaporation rates (12.28–8.37 kg/m^2^ h) and it could effectively collect them with high efficiencies (81–65% and 88–67%) in unmanned HNS trapping device. Our device described here can be specialized for collection of HNSs in layered oil/water mixture. Thus, further research is needed for other oil/water mixtures like suspension or emulsion. We believe that the novelty of our work will encourage other researchers to follow solar-driven oil/water separation method. Our HNS trapping device has also advantages in terms of unmanned and nonpowered systems, fast removal of HNSs, and effective collection of valuable HNSs.

## Data Availability

The data presented in this study are available on request from the corresponding author.

## Supplementary Materials

The following supporting information can be downloaded at: https://www.mdpi.com/article/10.3390/polym14030631/s1, Figures S1–S8: XRD data for the PUS/MNP/PVA. Images of PUS before and after dipping of PUS in toluene and xylene. Images and weight change data of PVA (MW: 130,000 Da, 1 wt%) film before and after dipping of the PVA film in toluene at 45 °C for 2 h. Images and weight change data of PVA (MW: 13,000 Da, 1 wt%) film before and after dipping of the PVA film in toluene at 45 °C for 2 h. Toluene evaporation rates of the PUS/MNP/LMW-PVA (MW: 13,000 Da) sample. Images of Al mirrors with different angles (90° ~ 160°). 90°, 100°, 115°, 130°, 145°, and 160°. Toluene evaporation rates of the Al mirror-equipped RPS.
