# Supplementary material for: Solar-Driven Unmanned Hazardous and Noxious Substance Trapping Devices Equipped with Reverse Piloti Structures and Cooling Systems
Source: Polymers (Basel). 2022 Feb 7;14(3):631. doi: 10.3390/polym14030631 (PMC8839944; doi:10.3390/polym14030631)
Supplement: Supplementary file 1 [file polymers-14-00631-s001.zip › polymers-1582503-supplementary.pdf]

Supporting Information

Solar-driven Unmanned Hazardous and Noxious Substance Trapping Devices Equipped with Reverse Piloti Structures and Cooling Systems

Ye Jin Kim [1], Hee Ju Kim [1], Yu Jin Seo [1], Ji Hee Choi [1], Hye Young Koo [2,*], and Won San Choi [1,*]

1 Department of Chemical and Biological Engineering, Hanbat National University, 125 Dongseodaero, Yuseong-gu, Daejeon 305-719, Republic of Korea; agoqkfkrl@naver.com (Y.J.K.); kimhj0924@naver.com (H.J.K.); 6285999@naver.com (Y.J.S.); wlgml1350@naver.com (J.H.C.)

2 Functional Composite Materials Research Center, Korea Institute of Science and Technology (KIST) Jeonbuk Institute of Advanced Composite Materials, 92 Chudong-ro, Bongdong-eup, Wanju-gun, Jeollabuk-do, Republic of Korea;

* Correspondence: choiws@hanbat.ac.kr (W.S.C.); koohy@kist.re.kr (H.Y.K.) Tel.:+82-42-821-1540 (Abbreviation Name),

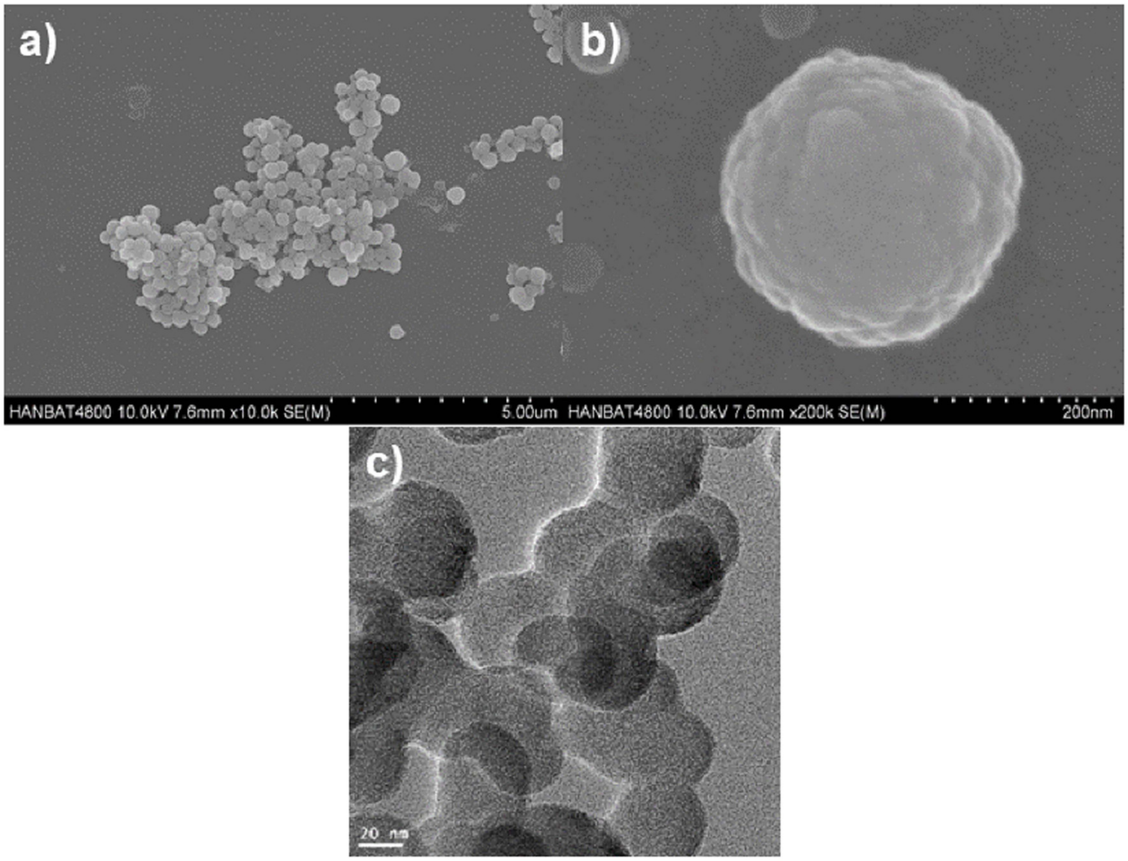

Figure S1. (a, b) SEM and (c) TEM images of MNPs and CS particles, respectively.

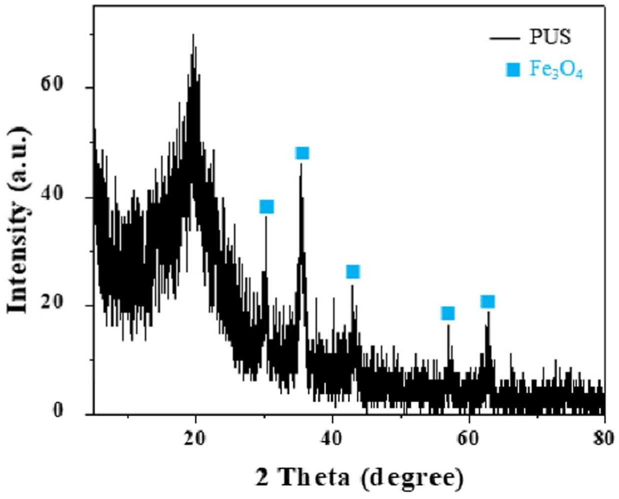

Figure S2. XRD data for the PUS/MNP/PVA.

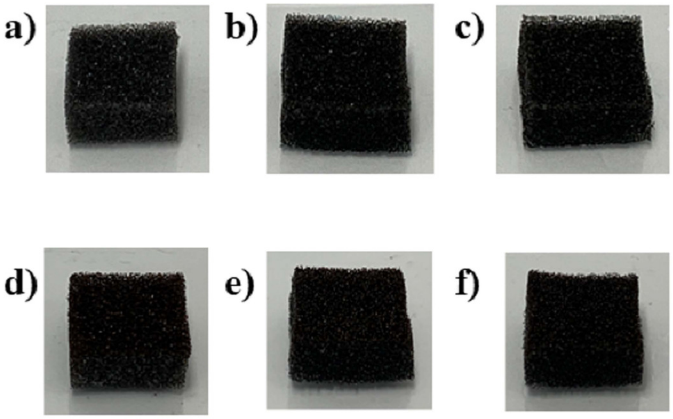

Figure S3. Images of PUS (a) before and (b, c) after dipping of PUS in (b) toluene and (c) xylene. Images of PUS/MNP/PVA (MW: 130,000) (d) before and (e, f) after dipping of PUS/MNP/PVA in (e) toluene and (f) xylene.

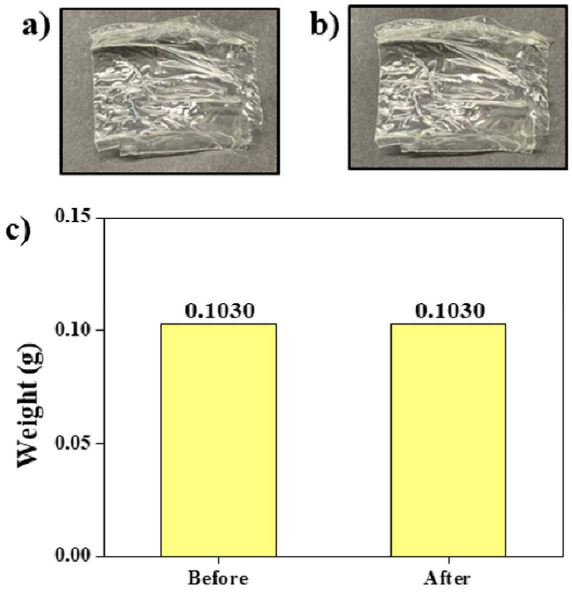

Figure S4. (a, b) Images and (c) weight change data of PVA (MW: 130,000 Da, 1 wt%) film (a) before and (b) after dipping of the PVA film in toluene at 45 °C for 2 h.

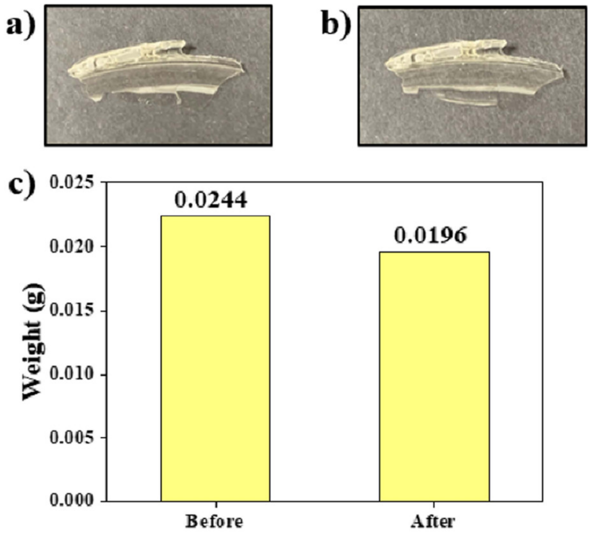

Figure S5. (a, b) Images and (c) weight change data of PVA (MW: 13,000 Da, 1 wt%) film (a) before and (b) after dipping of the PVA film in toluene at 45 °C for 2 h.

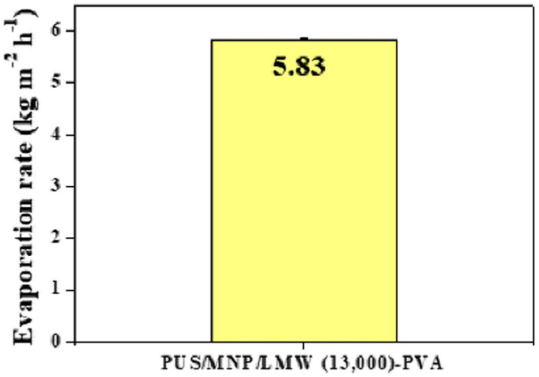

Figure S6. Toluene evaporation rates of the PUS/MNP/LMW-PVA (MW: 13,000 Da) sample.

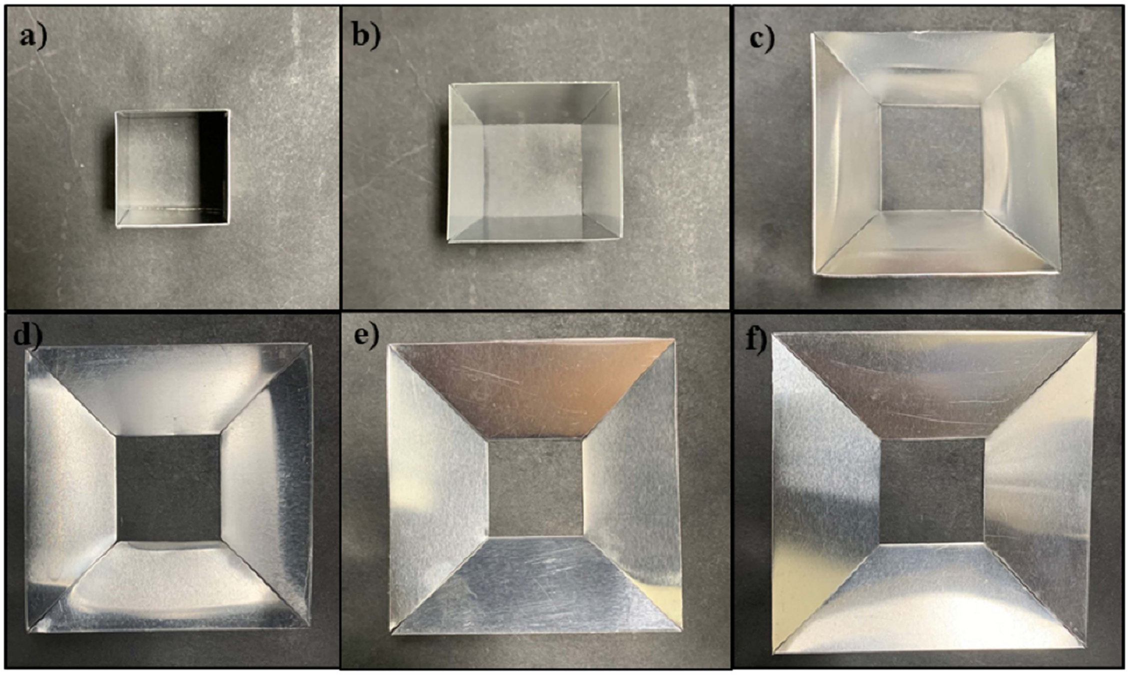

Figure S7. Images of Al mirrors with different angles (90° ~ 160°). (a) 90°, (b) 100°, (c) 115°, (d) 130°, (e) 145°, and (f) 160°.

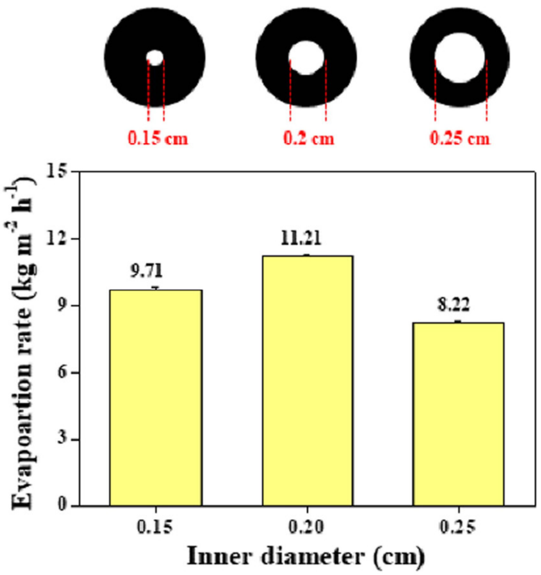

Figure S8. Toluene evaporation rates of the Al mirror-equipped RPS [PUS/MNP/HMW-PVA (MW: 130,000 Da)] with different hollow column ratios [30% (D: 0.15 cm), 40% (D: 0.2 cm), and 50% (D: 0.25 cm)
